# Development and Validation of a Novel UHPLC-MS/MS Method for the Quantification of Plinabulin in Plasma and Its Application in a Pharmacokinetic Study with Leukopenic Rats

**DOI:** 10.3390/ph16081153

**Published:** 2023-08-14

**Authors:** Xiaochen Niu, Dan Chen, Wei He, Yu Tang, Jianchun Zhao

**Affiliations:** 1 School of Medicine and Pharmacy, Ocean University of China, Qingdao 266003, China; 2 Marine Biomedical Research Institute of Qingdao, Qingdao 266073, China

**Keywords:** plinabulin, leukopenia, UHPLC-MS/MS, pharmacokinetics, dose proportionality

## Abstract

Plinabulin, a new antitumor drug developed from marine natural products that targets microtubules in cancer cells, is currently being tested in a phase III clinical study. Plinabulin has been clinically proven to be effective on leukopenia. However, to our knowledge, there are no reports investigating the pharmacokinetics of plinabulin in individuals with leukopenia and healthy individuals. In this study, we developed a rapid and sensitive UHPLC-MS/MS method for the detection of plinabulin for the first time. Using a novel cyclophosphamide-induced leukopenia model, we investigated the differences in the pharmacokinetic characteristics of plinabulin between rats with leukopenia and normal rats. Plinabulin and propranolol (IS) peaks were separated by gradient elution for a total run time of 5 min. The methodological validation showed a good accuracy (101.96–109.42%) and precision (RSD ≤ 5.37%) with the lower limit of quantification at 0.5 ng/mL. The recovery of plinabulin was between 91.99% and 109.75% (RSD ≤ 7.92%). The values of the area under the plasma concentration-time curve (AUC_0-t_) for leukopenia groups and control groups at doses of 0.5 mg/kg, 1 mg/kg, and 3 mg/kg were 148.89 ± 78.74 h·μg/L and 121.75 ± 31.56 h·μg/L; 318.15 ± 40.00 h·μg/L and 272.06 ± 42.85 h·μg/L; and 1432.43 ± 197.47 h·μg/L and 1337.12 ± 193.56 h·μg/L; respectively. The half-lives (t_1/2_s) of plinabulin were 0.49–0.72 h for leukopenia groups and 0.39–0.70 h for control groups at three doses, and the clearance rates (CLs) of plinabulin were 2.13–3.87 L/h/kg for leukopenia groups and 2.29–4.23 L/h/kg for control groups. Pharmacokinetic results showed that there was no significant pharmacokinetic difference between the normal group and the leukopenia group. Based on the power model, plinabulin exhibits a lack of dose proportionality over the dose range of 0.5–3 mg/kg after intravenous administration. This study provides guidance for the development of plinabulin as a potential candidate for the treatment of chemotherapy-induced leukopenia.

## 1. Introduction

**Figure 1:**
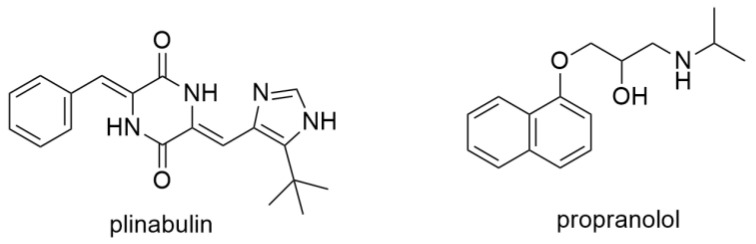
The chemical structures of plinabulin and propranolol.

Cancer is a major public health problem worldwide and has overtaken cardiovascular disease as the world’s first leading cause of death. In 2022, 1.92 million additional cancer cases and 0.61 million deaths were reported in the United States, among which leukopenia caused by cancer chemotherapy was the main cause of death [1,2]. Chemotherapy-induced neutropenia (CIN) increases the risk of infection and death in cancer patients. Granulocyte colony-stimulating factor (G-CSF) therapy is approved for the treatment of CIN, and non-G-CSF therapy is required to improve its efficacy and minimize side effects [3]. It has been reported that plinabulin (C_19_H_20_N_4_O_2_) (Figure 1) binds colchicine sites of βII more persistently and is a novel non-G-CSF selective immunoregulatory tubulin polymerization inhibitor with hematopoietic stem cell protective properties and anticancer effects [4,5]. It has been reported that plinabulin has important functions when acting with guanine nucleotide interchange factor stimulants and is mainly used in the treatment of neutropenia and non-small cell lung cancer [6]. Previous studies have shown that plinabulin is a potent vascular destabilizer, which can ameliorate leukopenia induced by multiple chemotherapies (including docetaxel, doxorubicin, and cyclophosphamide) through a different mechanism from G-CSF [5,7]. Currently, plinabulin is in phase III clinical trials for cancer and chemotherapy-induced neutropenia [8].

Although plinabulin is in phase III clinical trials, few references and preclinical data in vivo have been published [9]. Until now, only a preclinical study of MBRI-001, a deuterium-substituted plinabulin derivative, has been published [10]. MBRI-001 exhibits pharmacokinetic profiles quite different from plinabulin in vitro, and all the data of plinabulin are in vitro results [10]. All available reports are about plinabulin combined with other drugs, and all studies are based on health status [11]. It was reported that the pharmacokinetic profiles of many drugs varied between disease and non-disease states [12,13]. In pathological states, the metabolic enzymes of some drugs and the permeability of their cell membranes have been changed, and drug-related ADME (absorption, distribution, metabolism, and excretion) processes were altered, which led to changes in pharmacokinetic parameters [14]. However, to date, no pharmacokinetic studies of plinabulin in pathological states have been reported.

In the present study, a sensitive, reproducible, and practical method was developed for the determination of plinabulin in rat plasma and was used to investigate the pharmacokinetics of plinabulin in the leukopenia state for the first time. In addition, it is also the first work where dose proportionality was studied. This study might provide a theoretical basis for research on the development of plinabulin as a potential candidate for the treatment of leukopenia.

## 2. Results and Discussion

### 2.1. Experimental Design

To establish a model of leukopenia induced by chemotherapy, we chose an anticancer drug cyclophosphamide (CY) because of its inhibitory effect on bone marrow [15]. Rats were injected with 50, 70, or 90 mg/kg of CY on the first and third days, respectively. The leukocytes were counted by a hemocytometer as shown in Table 1. Compared with the control group, the white blood cell (WBC) counts for rats injected with different concentrations of CY were significantly decreased (*p* < 0.01). The level of WBC in the CY 70 mg/kg group was the second lowest among the three groups. If the WBC level is too low, it might lead to stopping radiotherapy and chemotherapy in clinical practice. Therefore, we finally chose 70 mg/kg as the experimental dose. This dose can significantly reduce the number of WBCs while keeping the experimental animals alive.

### 2.2. Optimization of UHPLC-MS/MS Method

**Figure 2:**
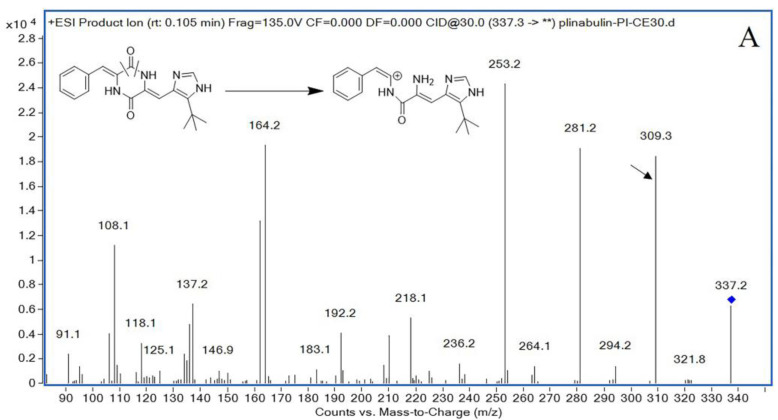

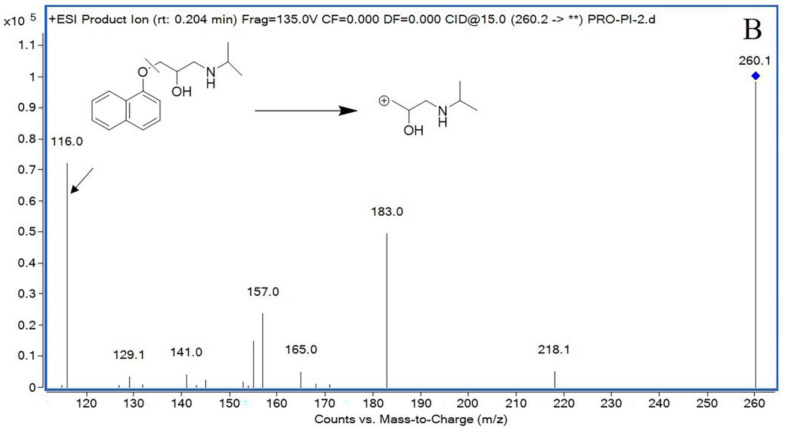
The mass spectra of plinabulin (**A**) and propranolol (**B**).

The UHPLC-MS/MS conditions were initially optimized for maximum sensitivity and resolution. In order to obtain good peak shape and chromatographic separation conditions, the composition of the mobile phase is particularly important. It was found that the addition of formic acid in the mobile phase could significantly improve peak shape and increase signal strength in our study. We tested different mobile phases, including water-acetonitrile and water-formic acid with different formic acid ratios. The addition of 0.2% formic acid to both mobile phases was found to be essential for promoting the ionization of plinabulin and internal standard (IS) propranolol (Figure 1) and could obtain good peak shape and high sensitivity. Both positive and negative ESI modes are used to optimize the mass response of plinabulin and IS, and it was found that the positive ESI mode has a higher response. In the positive ion mode, plinabulin and IS showed a protonated molecular ion at *m*/*z* 337.3 and 260.2 (Figure 2), respectively. Parameter optimization of mass spectrometer settings revealed the most abundant product ions were 309.3 for plinabulin at a collision energy of 22 V and 116.0 for IS at a collision energy of 15 V. Therefore, ion transitions for quantification were *m*/*z* 337.3 > 309.3 for plinabulin and *m*/*z* 260.2 > 116.0 for IS monitored using the multiple reaction monitoring (MRM) mode.

In the early stage of method development, there were two or three product ions that were used to monitor plinabulin and IS, respectively. After the method was determined, only one product ion displayed the best response in rat plasma. Usually, we believe that when the energy is constant, monitoring multiple ions simultaneously can have an impact on the response of each ion pair. There was no endogenous interference or other impurities. Therefore, only one product ion for quantification and identity confirmation, particularly, 309.3 for plinabulin and 116.0 for IS monitored using MRM mode, was enough. Using one ion pair for monitoring and quantification is common and has been previously reported [12,13].

### 2.3. Method Validation

**Figure 3:**
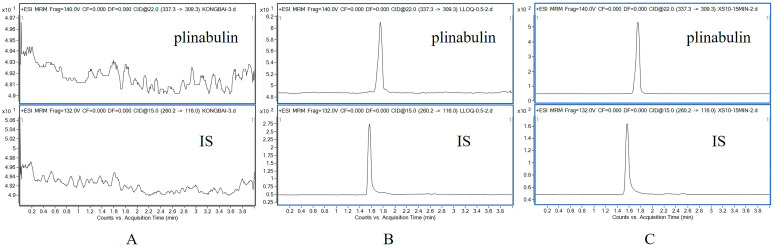
The typical MRM chromatograms of plinabulin and IS in Wistar rat plasma; (**A**) Wistar rat blank plasma; (**B**) blank rat plasma spiked with plinabulin and IS at LLOQ; (**C**) Wistar rat plasma samples collected at 15 min after intravenous injection of plinabulin (3 mg/kg).

Selectivity data are presented in Figure 3. We analyzed the chromatograms of blank rat plasma, blank rat plasma spiked with plinabulin at a lower limit of quantification (LLOQ), and rat plasma samples collected at 15 min after intravenous injection of plinabulin. The retention times of plinabulin and IS were 1.75 min and 1.56 min, respectively. We found no interference from endogenous plasma components or other impurities.

Based on 1/x^2^ weighted linear regression, the calibration curve of plinabulin was the best in the range of 0.5 ng/mL to 500 ng/mL. The typical standard curves of plinabulin in the plasma of Wistar rats are shown in Table 2. The sensitivity was assessed by adding plinabulin to LLOQ using plasma from six individual blank rats. Because there were no articles about the analytical method of plinabulin or similar compounds, we have not compared the LLOQ of our study with others. In general, LLOQ of 0.5 ng/mL is sensitive for bioanalytical methods [16,17,18,19,20]. In our study, the LLOQ was 0.5 ng/mL, and the value of the signal-to-noise ratio of LLOQ was greater than 10. We have not determined the limit of detection (LOD). The reasons are below: (1) In the pharmacokinetic study of plinabulin in our study, the concentrations below LLOQ have not been included in the calculation of pharmacokinetic parameters. Thus, the limit of detection (LOD) is not applied in our study. (2) The method was validated according to the guidelines of ICH M10 [21]. According to ICH M10, LOD is not included in the method validation.

The precision and accuracy data of the intra-day and inter-day are shown in Table 3. The inter-day precision of LLOQs was 15.95% and the intra-day precision of LLOQs was 9.42%. Relative standard deviations (RSD) of the low-quality control (LQC), middle-quality control (MQC), and high-quality control (HQC) were less than 7.62%, and the accuracy values were between 100.36% and 105.37%. All data were within acceptable requirements.

The stability analysis of plinabulin in Wistar rat plasma under different conditions is shown in Table 4. The stability under different conditions ranged from 89.23% to 110.58%, with RSD < 11.15%. There was no significant degradation of the plasma samples during treatment and storage.

To verify the dilution effect of plinabulin in Wistar rat plasma, samples were diluted 20-fold with blank rat plasma (Table 5). The RSD (%) and the relative error (RE) (%) ranges of the dilution methods were less than 15%, indicating that the dilution was in accordance with the standards.

The results of the matrix effect and recovery are shown in Table 6. The matrix effect of plinabulin ranged from 94.80% to 100.45% (RSD ≤ 11.28%). The recovery of plinabulin was between 91.99% and 109.75% (RSD ≤ 7.92%). All the RSD values conformed to the criteria. The results demonstrated that the matrix effect of the sample preparation method can be neglected and is not subject to endogenous interference.

At present, no analytical method has been developed for the determination of plinabulin. In this work, we developed a novel UHPLC-MS/MS method that provided a short analysis time of 5 min at a relatively low cost for the determination of plinabulin. Additionally, this assay exhibited good sensitivity, stability, the absence of a matrix effect, high recovery, and good dilution integrity.

### 2.4. Pharmacokinetics of Plinabulin in Leukopenic Rats

Leukopenia caused by bone marrow suppressive chemotherapy is a common side effect in the clinical treatment of cancer, which can increase the risk of infection in cancer patients [22]. Due to the reduction in leukocytes, the dose of chemotherapy may be reduced, leading to a delay in hospitalization and affecting the treatment effect. Depending on the extent and duration of their reduction, it increased the risk of febrile neutropenia infection [23]. However, the pharmacokinetic profiles could be changed in the leukopenia state [18]. Therefore, it is important to study the pharmacokinetics of plinabulin in the leukopenia state to provide more information for guiding clinical medication plans.

**Figure 4:**
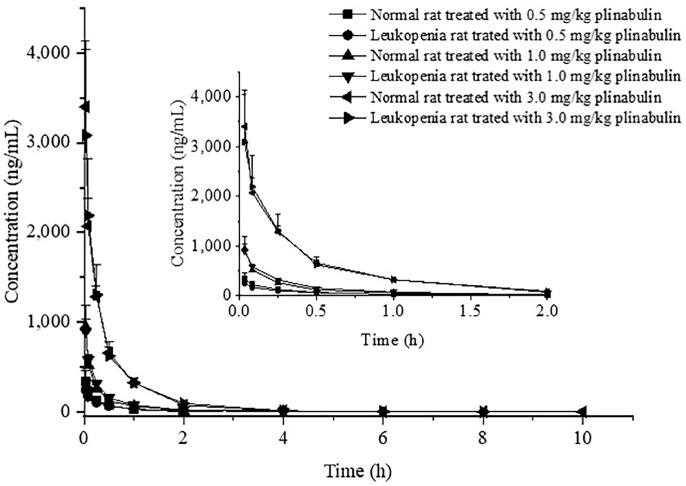
The integrated plasma concentration-time profiles of three dosages in each group (*n* = 6).

The mean plasma concentration-time profiles for plinabulin are presented in Figure 4. The main pharmacokinetic parameters are shown in Table 7. According to the results, we found that the pharmacokinetic characteristics of plinabulin with intravenous injection showed certain regularity. The results showed that the C_max_ (maximum concentration) and AUC (area under the plasma concentration-time curve) of plinabulin increased with increasing doses in the leukopenia and control groups at different doses. The half-lives (t_1/2_) of the leukopenia group and control group were 0.35–0.81 h, indicating that the elimination of plinabulin was very fast. The mean residence time (MRT) of plinabulin was short (0.35–0.64 h), indicating that plinabulin stayed in the rat body for a short time and was easy to be excreted. At the same dose, the pharmacokinetic parameters of plinabulin in the leukopenia group and control group showed little difference.

When two or more drugs are administered simultaneously or sequentially, the pharmacokinetic profiles of each may be altered due to drug–drug interactions. For example, the C_max_ and AUC of bedaquiline with the addition of rifabutin decreased by 38.8% and 65.5%, respectively, compared with those without rifabutin [24]. In recent years, more and more studies have been conducted on the pharmacokinetics of CY. Previous reports have shown that the C_max_ of CY is about 2 h after administration, and its half-life varies with different species [25]. In particular, the t_1/2_ of CY is close to 6 h in humans but less than 2 h in rats, cats, and dogs [26]. In this experiment, plinabulin was administered by intravenous injection 12 h after CY administration, with a half-life interval of more than five times. It means that the residual amount of CY in the body is low and its effect is negligible [26]. Thus, CY does not affect the pharmacokinetics of plinabulin. In our results, there was no significant difference in pharmacokinetics between the leukopenia group and the control group, which was in line with the expected results.

### 2.5. Dose Proportionality

**Figure 5:**
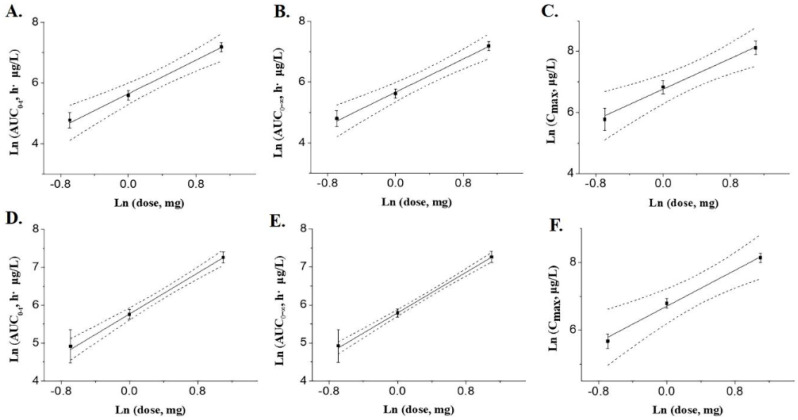
Relationship between systemic exposure and dose after intravenous administration of 0.5 to 3 mg/kg plinabulin in Wistar rats in the control groups and the leukocyte groups. Solid circles are mean observations, solid lines are fits based on power models, and dashed lines are 90% CI. (**A**) AUC_0–t_ in the control groups; (**B**) AUC_0–∞_ in the control groups; (**C**) C_2min_ in the control groups; (**D**) AUC_0–t_ in the leukocyte groups; (**E**) AUC_0–∞_ in the leukocyte groups; (**F**) C_2min_ in the leukocyte groups.

The method used to assess dose ratios from 0.5 to 3 mg/kg is to compare the 90% confidence interval (CI) of the slope (β1) with a modified acceptance range based on the power model [1 + (ln(θ_L_)/ln(r)), 1 + (ln(θ_H_)/ln(r)] [27]. The current FDA guidance defines θ_L_ = 0.80 and θ_H_ = 1.25, where θ_L_ and θ_H_ are the confidence intervals of the lower and upper limits, and r is the maximum dose ratio studied [27,28]. According to references [29,30,31], plots of the power model fit functions for C_2min_, AUC_0–t,_ and AUC_0–∞,_ and the associated 90% confidence intervals of the control and leukocyte groups are given in Figure 5. The results of the evaluation based on the power model are shown in Table 8. The slope (90% CI) of AUC_0-t_ for control groups was 1.386 (0.928, 1.844), the slope of AUC_0–∞_ was 1.370 (0.954, 1.787), and the slope (90% CI) was 1.252 (0.621, 1.884) for C_2min_. Using the same algorithm, the slope (90% CI) of AUC_0–t_ for leukopenia groups was 1.351 (1.124, 1.579), the slope of AUC_0–∞_ was 1.332 (1.197, 1.466), and the slope (90% CI) was 1.322 (0.673, 1.971) for C_2min_. The results of the statistical analysis showed that the 90% CIs for AUC_0–t_, AUC_0–∞,_ and C_2min_ of the control and the leukopenia groups spanned the acceptance interval (0.875,1.125) defined by the equations for r = 3/0.5, θ_L_ = 0.8, and θ_L_ = 1.25. Based on the criteria of the power model evaluation [27,32], it can be concluded that the results of the dose ratio for plinabulin intravenous administration showed inconclusive results in the dose range of 0.5–3 mg/kg.

## 3. Materials and Methods

### 3.1. Materials and Animals

Plinabulin (purity ≥ 98%) was purchased from Beijing Solarbio Technology Co., Ltd. (Beijing, China). Propranolol (purity ≥ 99%, internal standard, IS) was obtained from Sigma-Aldrich (St. Louis, MO, USA). HS-15 was purchased from Shanghai BASF Co., Ltd. (Shanghai, China), and propylene glycol was obtained from Beijing Fengli Jingqiu Pharmaceutical Co., Ltd. (Beijing, China) HPLC-grade acetonitrile and HPLC-grade methanol were purchased from Adamas-beta (Shanghai, China). HPLC-grade formic acid was from Fisher, USA. Cyclophosphamide (CY) was purchased from Sinopharm Chemical Reagent Co., Ltd. (Shanghai, China). Deionized water was from Watsons professional drinking water.

Forty-eight Wistar rats (males, weight 180 ± 20 g) were purchased from Jinan Pengyue Experimental Animal Breeding Co., Ltd. (Shandong, China). The experimental animal production license number is SCXK (Lu) 2022006. All Wistar rats were maintained in the SPF animal room with a temperature of 22 ± 2 °C and a humidity of 60 ± 5% for 12 h light-dark cycles. Animals were allowed to take in rodent food and drink water freely before intravenous administration. The animal study protocol was approved by the Animal Experimental Ethical Committee of Ocean University of China (Permit NO. OUC-AE-2022-109, and the date of approval was 7 March 2022).

### 3.2. Methods

#### 3.2.1. Establishment of the Leukopenia Rat Model

On the first and third days of the experiment, rats were intraperitoneally injected with the anticancer drug CY at 50, 70, and 90 mg/kg, respectively, to select the appropriate dose for establishing the leukopenia model. Meanwhile, rats in the control group were intraperitoneally injected with the same volume of saline. Blood (20 μL) was collected at 12 h after the last injection, and the white blood cells (WBCs) were counted by an IDEXX ProCyte Dx Hematology Analyzer (Shanghai IDEXX Laboratories Co., Ltd.) (Shanghai, China).

#### 3.2.2. UHPLC-MS/MS Assay

Sample analysis was performed on an Agilent 1290 system consisting of a G7120A high-speed pump, a G7167B auto-sampler, and a G7116B MCT column oven. Plinabulin and IS were detected by Agilent G6460C electrospray ionization (ESI) triple mass spectrometry. The chromatographic column was Capcell Pak C18 (2.0 × 50 mm, 5 μm) at 30 °C. The mobile phase consisted of a mixture of water (A, containing 0.2% (*v*/*v*) formic acid) and acetonitrile (B, containing 0.2% (*v*/*v*) formic acid) at a flow rate of 0.4 mL/min with a gradient elution procedure as follows: 0–0.5 min, 5% B; 0.5–2.0 min, 5–35% B; 2.0–3.0 min, 35–95% B; 3.0–3.5 min, 95% B; 3.5–3.8 min, 95–5% B; 3.8–5.0 min, 5% B. The volume of injection was 2 μL, and the sample temperature was maintained at 20 °C.

The mass spectrometer was set in a positive ionization pattern under multiple reaction monitoring (MRM) mode, and the optimized mass spectrum conditions were as follows: gas temperature: 350 °C; gas flow rate: 11 L/min; sprayer: 30 psi; and capillary voltage: 4 kV. The fragmentor was optimized to 140 V for plinabulin and 132 V for IS. Plinabulin and IS had collision energies of 22 V and 15 V, respectively. The precursor ion and product ion were *m*/*z* 337.3 → 309.3 for plinabulin, and *m*/*z* 260.2 → 116.0 for IS, respectively.

#### 3.2.3. Standard and Quality Control (QC) Sample Preparations

Working solutions and QC solutions for plinabulin were prepared by dissolving plinabulin into a freshly prepared stock solution of 10 mg/mL with DMSO (dimethyl sulfoxide) and serially diluting the stock solution with 60% acetonitrile. The most ideal internal standard should be the isotopically labeled plinabulin compound. Due to the double bond in the structure of plinabulin, cis-trans isomerization occurs easily under light conditions [33]. Isomerization also occurs in isotopically labeled plinabulin and these isomeric compounds are not stable [34]. Due to its high stability, propranolol has been widely used as a universal internal standard in the analysis of biological samples [35,36,37,38,39]. Hence, to eliminate the systematic error of plinabulin content determination, propranolol was chosen as IS for quantification. Besides high stability, propranolol presents chromatographic properties similar to plinabulin, reinforcing its application as IS in the present study. Propranolol solution with a concentration of 20 ng/mL was prepared with acetonitrile and stored at 4 °C until further analysis.

Ten calibration samples and QC samples were prepared by adding 10 μL of a 10-fold concentration of calibrators to 100 μL blank rat plasma. The mixture was vortexed for 30 s, and concentrations of the ten calibration samples of plinabulin in plasma were 0.5, 1, 2, 5, 10, 20, 50, 100, 200, and 500 ng/mL, respectively. QC samples were prepared at four concentrations of 0.5 ng/mL (lower limit of quantification, LLOQ), 1 ng/mL (low-quality control, LQC), 100 ng/mL (middle-quality control, MQC), and 400 ng/mL (high-quality control, HQC) with independent preparation, and then 200 μL acetonitrile containing IS (20 ng/mL) was added to precipitate protein. After vortexing for 1 min and centrifugation at 14,000 rpm for 10 min at 4 °C, 100 μL supernatant was transferred into the auto-sampler vials for UHPLC-MS/MS analysis with 2 μL injection volume.

#### 3.2.4. Plasma Sample Preparation

All rat plasma samples were thawed at room temperature before analysis. To a 100 μL plasma sample was added 10 μL 60% acetonitrile, then 200 μL acetonitrile containing IS (20 ng/mL) was added to precipitate protein. The mixture was vortex-mixed for 1 min and centrifuged at 14,000 rpm for 10 min at 4 °C. Finally, 100 μL supernatant was collected into the auto-sampler vials for UHPLC-MS/MS analysis with 2 μL injection volume.

#### 3.2.5. Method Validation

The method was validated according to the guidelines of ICH M10 Bioanalytical Method Validation and Study Sample Analysis (24 May 2022) [21].

The selectivity of plinabulin was determined by analyzing the blank rat plasma spiked with plinabulin and IS and rat plasma collected at 15 min after intravenous administration of a 3 mg/kg dose of plinabulin to explore the potential endogenous interference between analytes.

Linearity was evaluated by plotting the plinabulin/IS response ratios versus the concentrations of plinabulin. The calibration curves ranged from 0.5 to 500 ng/mL with ten points fitted y = bx + c by weighting factor 1/x^2^. Every standard curve solution was freshly prepared in every validation and sample detection. The calibration curves were accepted if R^2^ > 0.99 (R^2^, coefficient of determination). The sensitivity was evaluated by LLOQ samples, which was the lowest calibration curve concentration. The RE of LLOQ should be within ±20%, and RSD should not exceed 20%. The signal-to-noise ratio (S/N) was >10.

The accuracy and precision were estimated by six replicates of LLOQ, LQC, MQC, and HQC samples (0.5, 1, 100, and 400 ng/mL, respectively) in intra- and inter-day batches. The accuracy and precision of the inter-day measurements were assessed by investigating three batches of LLOQ, LQC, MQC, and HQC samples on three continuous days. Precision was expressed as a percentage (%) of RSD. The accuracy should be within ±15% for QC samples and within ±20% for LLOQ. The precision should be within 15% for QC samples and within 20% for LLOQ.

The dilution integrity of the bioassay was determined by using blank Wistar rat plasma diluted 20-fold. The RE % and RSD % should be less than 15%.

The extraction recovery was determined by comparing the peak area of plinabulin in the supernatant of rat blank plasma with that of precipitated blank plasma at three QC levels, and each QC sample was set up in 6 replicates. The RSD (%) of the peak area ratio should be less than 15%. The matrix effect was determined by determining the peak area of plinabulin in the supernatant of the precipitated blank rat plasma by acetonitrile (*n* = 6) with those in 60% acetonitrile at three QC levels. The RSD (%) of the peak area ratio should be less than 15%.

The stability of plinabulin in rat plasma was evaluated by analyzing six replicates of plasma samples at LQC and HQC levels under different storage conditions. The short-term stability was assessed by analyzing the samples in plasma at room temperature for 6 h. LQC and HQC samples were stored at −80 °C for 28 consecutive days to assess long-term stability. The stability of the freeze–thaw cycle was performed by analyzing samples in plasma through three freeze–thaw cycles from −80 °C to room temperature. The stability of processed samples in the auto-sampler was evaluated after maintaining post-preparative samples in the auto-sampler for 24 h. The stability of the samples was evaluated by comparing them with corresponding spiked concentrations of analytes. The RE should be within ±15%, and RSD < 15%.

#### 3.2.6. Pharmacokinetics Study

Thirty-six Wistar rats (18 normal rats and 18 leukopenia rats) were randomly divided into six groups (*n* = 6 per group). They received 0.5, 1, or 3 mg/kg plinabulin intravenously, respectively. The rats were given free water before intravenous administration. A single dose of plinabulin was administered to each group and an equivalent of 0.3 mL blood sample was collected into heparinized Eppendorf tubes by post-orbital venous sinus before dosing and subsequently at 2 min, 5 min, 15 min, 0.5 h, 1 h, 2 h, 4 h, 6 h, 8 h, and 10 h after intravenous administration. Blood samples were centrifuged at 8000 rpm for 10 min at 4 °C, and the supernatants were collected. The plasma samples were stored at −20 °C until further analysis. A 100 μL plasma sample was added with 10 μL 60% acetonitrile, then 200 μL acetonitrile containing IS (20 ng/mL) was added to precipitate protein. The mixture was vortex-mixed for 1 min and centrifuged at 14,000 rpm for 10 min at 4 °C. Finally, the supernatants were collected into the auto-sampler vials for UPLC–MS/MS analysis.

### 3.3. Statistical Analyses

All values were expressed as mean ± standard deviation. Statistical analysis was calculated by Microsoft Office Excel 2016. A difference at *p* < 0.05 was considered to be statistically significant (marked as *). The higher significance level was set at *p* < 0.01 (marked as **). The mean plasma concentration-time curves were drawn by Origin 8.0. The pharmacokinetic parameters were analyzed by DAS 3.0 (BioGuider Co., Shanghai, China) based on non-compartmental analysis. The dose ratios were assessed by fitting the relationship of the ln-transformed dose-normalized parameters to the ln-transformed dose using a linear regression model [29]. The value of the constant of proportionality β1 and its corresponding 90% confidence interval (CI) were estimated using Origin 8.0 software. Dose proportionality was concluded if the plot of pharmacokinetic parameter vs. dose indicated linearity (β1 = 1) and the 90% CI for the slope fell into the acceptance range [27,40].

## 4. Conclusions

In conclusion, we have developed a sensitive, reproducible, and practical UHPLC-MS/MS method for the determination of plinabulin in rat plasma and fully validated the method for the first time. The protein precipitation method was simple and acceptable for the recovery of plinabulin. The calibration curve of plinabulin was established in the range of 0.5 (LLOQ) to 500 ng/mL with reproducible intra- and inter-day accuracy and precision. Our UHPLC-MS/MS method has a high sensitivity, a good resolution, a moderate price, and is quite suitable for accurate quantification of plinabulin in pharmacokinetic studies in rats. In this study, we have compared the pharmacokinetic characteristics of plinabulin in normal and leukopenia conditions. The results showed that the pharmacokinetic parameters such as C_2min_ and AUC of plinabulin appeared to be proportional to the dose administered, although they did not meet the statistical criteria for dose proportionality. We did not find any pharmacokinetic difference in plinabulin between the leukopenic and normal groups. It provided some references for the next research and clinical development of plinabulin. Limited by our experimental conditions and other uncontrollable factors, we were unable to explore the detailed mechanism in our study. Next, we plan to study the detailed mechanism of plinabulin pharmacokinetic characteristics in terms of drug–drug interactions and pathological changes in the disease state. We will also explore the relationship between its pharmacodynamics and pharmacokinetics to provide better guidance on the clinical use of plinabulin.

## Figures and Tables

**Table 1:** The levels of white blood cells of blank rats and rats treated with CY (*n* = 6).

Group	Numbers	WBCs (10^9^/L)
Control	6	10.19 ± 4.42
CY 50 mg/kg	6	3.40 ± 0.67 **
CY 70 mg/kg	6	2.02 ± 0.64 **
CY 90 mg/kg	6	1.29 ± 0.18 **

** *p* < 0.01 vs. control.

**Table 2:** The typical standard curves of plinabulin (*n* = 3) in rat plasma.

Batch	Intercept	Slope	R^2^
1	0.041614	0.979321	0.9969
2	0.018414	0.912291	0.9962
3	0.016761	0.883853	0.9967

**Table 3:** The intra- and inter-day precision and accuracy for plinabulin in rat plasma.

Spiked Concentration (ng/mL)		Measured Concentration (ng/mL) (Mean ± SD, *n* = 6)			Overall Mean	Intra-Day	Inter-Day	RE
		Batch 1	Batch 2	Batch 3	(ng/mL)	RSD (%)	RSD (%)	(%)
LLOQ	0.5	0.49 ± 0.02	0.54 ± 0.05	0.55 ± 0.07	0.53	9.42	15.95	5.37
LQC	1	1.02 ± 0.07	1.05 ± 0.10	1.07 ± 0.06	1.04	7.62	5.82	4.41
MQC	100	100.15 ± 4.88	105.90 ± 2.83	105.29 ± 1.37	103.78	3.23	7.45	3.78
HQC	400	396.53 ± 10.40	399.99 ± 8.66	407.83 ± 1.79	401.45	1.96	3.53	0.36

**Table 4:** Stability of plinabulin in rat plasma under various storage conditions (*n* = 6).

Stability Conditions	Concentration (ng/mL)	Mean ± SD (ng/mL)	RSD (%)	RE (%)
Room temperature for 6 h	1	1.05 ± 0.07	6.43	5.45
	400	395.94 ± 11.96	3.02	−1.01
Long-term for 28 days (−80 °C)	1	1.11 ± 0.12	11.15	10.58
	400	356.94 ± 12.02	3.37	−10.77
Three freeze–thaw cycles	1	1.01 ± 0.07	6.85	1.40
	400	411.99 ± 12.11	2.94	3.00
Auto-sampler for 24 h (10 °C)	1	1.07 ± 0.06	5.44	7.46
	400	419.94 ± 13.50	3.21	4.99

**Table 5:** The results of dilution integrity (*n* = 6).

Concentration (ng/mL)	Dilution Ratios	Mean ± SD	RSD (%)	RE (%)
5000	20	4872.28 ± 79.30	1.63	−2.55

**Table 6:** Matrix effect and recovery of plinabulin in rat plasma (*n* = 6).

Concentration (ng/mL)	Matrix Effect		Recovery Rate	
	Mean ± SD (%)	RSD (%)	Mean ± SD (%)	RSD (%)
1	94.80 ± 9.70	10.23	100.63 ± 7.97	7.92
100	100.45 ± 3.04	3.03	109.75 ± 4.50	4.10
400	97.53 ± 11.00	11.28	91.99 ± 2.87	3.12

**Table 7:** Pharmacokinetic parameters of plinabulin in normal and leukopenic rats (Mean ± SD, *n* = 6).

Parameters	Normal Rats			Leukopenic Rats		
	0.5 mg/kg	1 mg/kg	3 mg/kg	0.5 mg/kg	1 mg/kg	3 mg/kg
AUC_0–t_ (h·μg/L)	121.75 ± 31.56	272.06 ± 42.85	1337.12 ± 193.56	148.89 ± 78.74	318.15 ± 40.00	1432.43 ± 197.47
AUC_0–∞_ (h·μg/L)	124.755 ± 32.76	278.94 ± 43.15	1337.62 ± 193.49	149.80 ± 79.09	327.56 ± 35.40	1432.97 ± 197.55
t_1/2_ (h)	0.39 ± 0.04	0.41 ± 0.03	0.70 ± 0.12	0.57 ± 0.29	0.49 ± 0.04 *	0.72 ± 0.09
CL (L/h/kg)	4.23 ± 1.05	3.66 ± 0.54	2.29 ± 0.35	3.87 ± 1.31	3.08 ± 0.34	2.13 ± 0.33
Vd (L/kg)	2.37 ± 0.58	2.14 ± 0.35	2.30 ± 0.45	2.75 ± 0.38	2.19 ± 0.39	2.19 ± 0.26
MRT (h)	0.40 ± 0.05	0.38 ± 0.03	0.58 ± 0.05	0.86 ± 0.75	0.45 ± 0.06	0.60 ± 0.04
C_2min_	340.30 ± 122.59	948.06 ± 233.85	3400.53 ± 735.75	298.41 ± 56.52	900.05 ± 129.18	3443.13 ± 420.59

* *p* < 0.05 vs. normal rats.

**Table 8:** Assessment of dose proportionality of plinabulin based on the power model.

Groups	Parameters	Acceptance Range	β1	90% Confidence Interval
Control groups	AUC_0–t_	0.875–1.125	1.386	0.928–1.844
	AUC_0–∞_	0.875–1.125	1.370	0.954–1.787
	C_2min_	0.875–1.125	1.252	0.621–1.884
Leukopenia groups	AUC_0–t_	0.875–1.125	1.351	1.124–1.579
	AUC_0–∞_	0.875–1.125	1.332	1.197–1.466
	C_2min_	0.875–1.125	1.322	0.673–1.971

## Data Availability

Data are contained within the article.
